# Unveiling the ecological significance of phosphorus fractions in shaping bacterial and archaeal beta diversity in mesotrophic lakes

**DOI:** 10.3389/fmicb.2023.1279751

**Published:** 2023-10-11

**Authors:** Haijun Yuan, Runyu Zhang, Qiuxing Li, Qiao Han, Qiping Lu, Jing Wu

**Affiliations:** ^1^State Key Laboratory of Environmental Geochemistry, Institute of Geochemistry, Chinese Academy of Sciences, Guiyang, China; ^2^University of Chinese Academy of Sciences, Beijing, China; ^3^College of Earth Science, Chengdu University of Technology, Chengdu, China

**Keywords:** bacteria, archaea, beta diversity, turnover, nestedness, phosphorus fractions, mesotrophic lakes

## Abstract

Both community variation and phosphorus (P) fractions have been extensively studied in aquatic ecosystems, but how P fractions affect the mechanism underlying microbial beta diversity remains elusive, especially in sediment cores. Here, we obtained two sediment cores to examine bacterial and archaeal beta diversity from mesotrophic lakes Hongfeng Lake and Aha Lake, having historically experienced severe eutrophication. Utilizing the Baselga’s framework, we partitioned bacterial and archaeal total beta diversity into two components: species turnover and nestedness, and then examined their sediment-depth patterns and the effects of P fractions on them. We found that total beta diversity, species turnover or nestedness consistently increased with deeper sediment layers regarding bacteria and archaea. Notably, there were parallel patterns between bacteria and archaea for total beta diversity and species turnover, which is largely underlain by equivalent processes such as environmental selection. For both microbial taxa, total beta diversity and species turnover were primarily constrained by metal oxide-bound inorganic P (NaOH-Pi) and sediment total phosphorus (STP) in Hongfeng Lake, while largely affected by reductant-soluble total P or calcium-bound inorganic P in Aha Lake. Moreover, NaOH-Pi and STP could influence bacterial total beta diversity by driving species nestedness in Hongfeng Lake. The joint effects of organic P (Po), inorganic P (Pi) and total P fractions indicated that P fractions are important to bacterial and archaeal beta diversity. Compared to Po fractions, Pi fractions had greater pure effects on bacterial beta diversity. Intriguingly, for total beta diversity and species turnover, archaea rather than bacteria are well-explained by Po fractions in both lakes, implying that the archaeal community may be involved in Po mineralization. Overall, our study reveals the importance of P fractions to the mechanism underlying bacterial and archaeal beta diversity in sediments, and provides theoretical underpinnings for controlling P sources in biodiversity conservation.

## Introduction

Biodiversity has potentially dramatic effects on aquatic (Zhang et al., 2021; Wang et al., 2021a) or terrestrial (Delgado-Baquerizo et al., 2020; Zhao et al., 2022) ecosystem functioning and stability. Prior studies have documented that ecosystems containing more species exhibit higher levels of ecosystem functions (Xun et al., 2019). Especially in naturally assembled communities, high biodiversity shows a strong positive effect on multiple ecosystem functions such as biomass production and trophic interactions (van der Plas, 2019). More importantly, the ongoing biodiversity loss is dramatically weakening the functioning of ecosystems. As such, diversity-triggered deterministic assembly processes are critical to understanding the degradation or enhancement in ecosystem functioning (Moroenyane et al., 2016; Xun et al., 2019). In recent decades, untangling the underlying mechanisms of biodiversity or biogeographic patterns has been considered as a central topic in ecology and microbial ecology (Zhou and Ning, 2017; Wang W. et al., 2020). There is increasing evidence that microbial alpha diversity has a crucial role in ecosystem function or services, but less attention has been paid to beta diversity (i.e., community variation between biological composition) (Xun et al., 2019). Beta diversity bridges the gap between local (alpha) and regional (gamma) diversity and is often applied to quantify the variation of species compositions between different ecological communities (Whittaker, 1960). Particularly, beta diversity interacts with alpha-diversity gradients, both resulting from community assembly via local or regional environmental filters (Soininen et al., 2018). Moreover, beta diversity, in contrast to alpha diversity, can better capture spatial or temporal dynamics of biodiversity patterns. Considering beta diversity in landscape-scale conservation (also known as ecosystem approach, can bring huge benefits to biodiversity and economy at large landscape scales) efforts will greatly contribute to improving ecosystem function, particularly in abiotically heterogeneous landscapes (van der Plas et al., 2023). In aquatic or terrestrial ecosystems, microbes, as key factors driving biogeochemical cycling processes, have a critical role in ecosystem functioning and services and are fundamental in maintaining ecosystem stability (Singh et al., 2010; Wu et al., 2019). Meanwhile, such element cycles release large amounts of reactive nitrogen (N), phosphorus (P) and heavy metals, leading to dramatic growth of aquatic plants and phytoplankton that consume excessive nutrients dissolved oxygen and light intensity. Accordingly, aquatic ecosystems and biodiversity therein are substantially threatened by environmental stressors like eutrophication or chemical pollution (Xiong et al., 2020). Given that such relationship among biodiversity and ecosystem functioning is scale-dependent (van der Plas et al., 2023), examining microbial beta diversity along environmental gradients, such as sediment depth (Yuan et al., 2021), is essential for understanding the variations in ecosystem functioning and stability.

It has long been the core of community ecology for unraveling the driving mechanisms of ecological communities, reflecting that beta diversity plays a pivotal role in community assemblage (i.e., the mechanisms underlying biodiversity organization) (Mori et al., 2018). More recently, from micro- to macro-organisms, beta diversity has been extensively partitioned into components turnover and nestedness to examine the ecological processes that shape community structures (Marta et al., 2021). Based on Sørensen dissimilarity index, Baselga (2010) proposed a framework to decompose beta diversity into turnover (i.e., species replacement) and nestedness, which provides valuable insights into biodiversity effects. The former reflects the replacement between species without changing species richness, whereas the latter indicates the richness differences caused by non-random species gain and loss (Baselga, 2010). Partitioning beta diversity may yield additional insights into biodiversity effects such as selection and complementarity effects (Mori et al., 2018), which identify the possible contributions of the turnover or nestedness components, thereby revealing whether community variation result from species replacement and species gain or loss. Compared to traditional beta diversity index like Bray–Curtis dissimilarity, partitioning beta diversity can provide a valuable fresh perspective to address the underlying causes of community variation in response to environmental changes (Viana et al., 2016; García-Navas et al., 2022). Specifically, phosphorus, as a limiting nutrient, acts as environmental filtering to constrain biological succession (Zheng et al., 2021) and can also drive community variation by influencing species turnover or nestedness. For example, species turnover dominates bacterial total beta diversity in freshwater ecosystems, but is largely constrained by water total P, substantially altering the water-depth pattern of bacterial community variation (Wu et al., 2020; Yuan et al., 2022). Therefore, partitioning beta diversity may help disentangle the ecological mechanisms governing microbial community variation under P limitation.

Currently, phosphorus is recognized as a primary limiting nutrient element in both aquatic and terrestrial ecosystem productivity (Cramer, 2010) and poses a considerable threat to biodiversity conservation (Ceulemans et al., 2014). A growing body of evidence suggests that phosphate (PO_4_^3−^) or total phosphorus can essentially alter the structure of bacterial, archaeal and fungal communities in aquatic ecosystems (Zhang et al., 2021; Guo et al., 2022). However, the underlying mechanisms of microbial beta diversity under the influence of P fractions are, hitherto, underexplored, especially in sediment cores. According to the Psenner fractionation method (Psenner et al., 1988), P fractions are primarily classified into five categories: loosely adsorbed P (NH_4_Cl-P), reductant-soluble P (BD-P), metal oxide-bound P (NaOH-P), calcium-bound P (HCl-P) and residual P (Res-P). Such P fractions can effectively distinguish the phosphorus sources buried in sediments and thereby disentangle the release mechanism of endogenous P in lakes or reservoirs (Zhu et al., 2013). Typically, such potentially mobile phosphorus can be well-applied to evaluate the mechanism underlying endogenous P released from sediments in lake ecosystems (Rydin, 2000). More recently, such relationship between P fractions and microbes is widely concerned in terrestrial ecosystems (Yang et al., 2023), but it is still largely missing in aquatic ecosystems. For example, microbial population have a crucial role in forming or releasing soil labile phosphorus across Tibetan alpine grasslands (Li et al., 2022), while P fractions can also influence the abundance of *Actinomycetes* by governing the enrichment of soil organic matter (Bian et al., 2022). Especially in rhizosphere soils, calcium-bound P can effectively inhibit the increase of microbial composition and biomass P (Teng et al., 2018). Conversely, in aquatic ecosystems, both mobile P and calcium-bound P are positively correlated with bacterial phyla such as *Firmicutes* and *Proteobacteria* (Yin et al., 2022). Notably, BD-P and NaOH-P are available and released easily from sediments, largely accelerating the eutrophication processes of water bodies and promoting the growth of phytoplankton (Cavalcante et al., 2018). Previous studies have mostly focused on the importance of total phosphorus to microbial communities, largely neglecting the contribution of specific components contained in total phosphorus. Dividing total phosphorus into various forms (i.e., specific P components) of P is an effective method to study the compositions and properties of sediment P (Yin et al., 2022). Intriguingly, these sediment P fractions are closely related to the endogenous release mechanism of lake eutrophication (Zhu et al., 2013). Additionally, partitioning beta diversity into species turnover and nestedness can well reveal the succession mechanism underlying microbial communities (Marta et al., 2021). Correspondingly, based on the effects of phosphorus components on microbial beta diversity, it is not difficult to infer whether lake eutrophication poses a threat to microbial communities. As such, coupling P fractions with beta diversity partition may provide a better way to unravel the driving mechanisms underlying microbial community variation.

Here, we obtained two sediment cores from Hongfeng Lake and Aha Lake, both of which are located in the Yunnan-Guizhou Plateau, Southwest China. Bacterial and archaeal communities were then quantified with the sequencing data of the *16S rRNA* gene. According to Baselga’s framework, we partitioned total beta diversity into turnover and nestedness, and then explored their sediment-depth patterns regarding bacteria and archaea. Further, we conducted in-depth analyses to uncover the relationships between beta diversity and P fractions. We primarily focused on three objectives: (1) to elucidate the mechanisms underlying bacterial or archaeal beta diversity along sediment depth and determine how turnover and nestedness essentially contribute to total beta diversity, (2) to reveal such cross-taxon relationship between bacteria and archaea in terms of beta diversity components, and (3) to identify the importance of each P fraction to microbial beta diversity and further evaluate how organic, inorganic and total P fractions drive bacterial and archaeal beta diversity.

## Materials and methods

### Study area and field sampling

The studied lakes, Hongfeng Lake and Aha Lake, have high internal P loading in sediments and are located in the karst region of the Yunnan-Guizhou Plateau, Southwest China. Hongfeng Lake, also known as Hongfeng Reservoir, has a surface area of 57.2 km^2^ with a maximum water depth of 45 m and holds 601 million m^3^ of lake water (Wang et al., 2016a). Hongfeng Lake is a typical P-limited artificial lake, showing a total P concentration of 0.03–0.10 mg L^−1^ and a TN/TP ratio of ~30 (Zhu et al., 2013). The total P content in sediments ranges from 766 to 4,306 mg kg^−1^, with an average of 1815 mg kg^−1^ (Wang et al., 2015). In 1978, Hongfeng Lake began to serve as a fish farm for economic benefit (Chen et al., 2019), which largely promoted the transition of its trophic status from oligotrophic to eutrophic (Yu et al., 2022). In particular, due to large amounts of P released from fish food and excrement, Hongfeng Lake experienced severe eutrophication and algal blooms in the mid-late 1990s. Notably, algal blooms occur frequently in Hongfeng Lake even though external phosphorus loading is well controlled in recent decades (Ma et al., 2022), presumably owing to the internal phosphorus loading released from sediments.

Aha Lake, constructed or initially impounded in 1960, is a typical artificial lake and situated in the suburb of Guiyang City, the capital of Guizhou Province (Chen et al., 2015). Aha Lake shows a total watershed area of 190 km^2^ and a capacity of 542 million m^3^ and is mainly recharged by five tributaries such as the Youyu, Baiyan, Caichong, Lannigou and Sha Rivers (Ni et al., 2021). Notably, it covers a surface area of 4.5 km^2^ and has a maximum water depth of 26 m (Chen et al., 2015). Aha Lake is a P-limited and deep plateau lake (Liu et al., 2019), with total P concentrations ranging from 0.022 to 0.205 mg L^−1^ and an average of 0.046 mg L^−1^ (Wang et al., 2021b). Owing to the aggravation of anoxia in the hypolimnion, large amounts of pollutants like phosphorus have been released from the sediments (Lan et al., 2017). Such phosphorus can broadly induce algal blooms and thereby endanger the water quality of this lake (Han et al., 2018).

**Figure 1:**
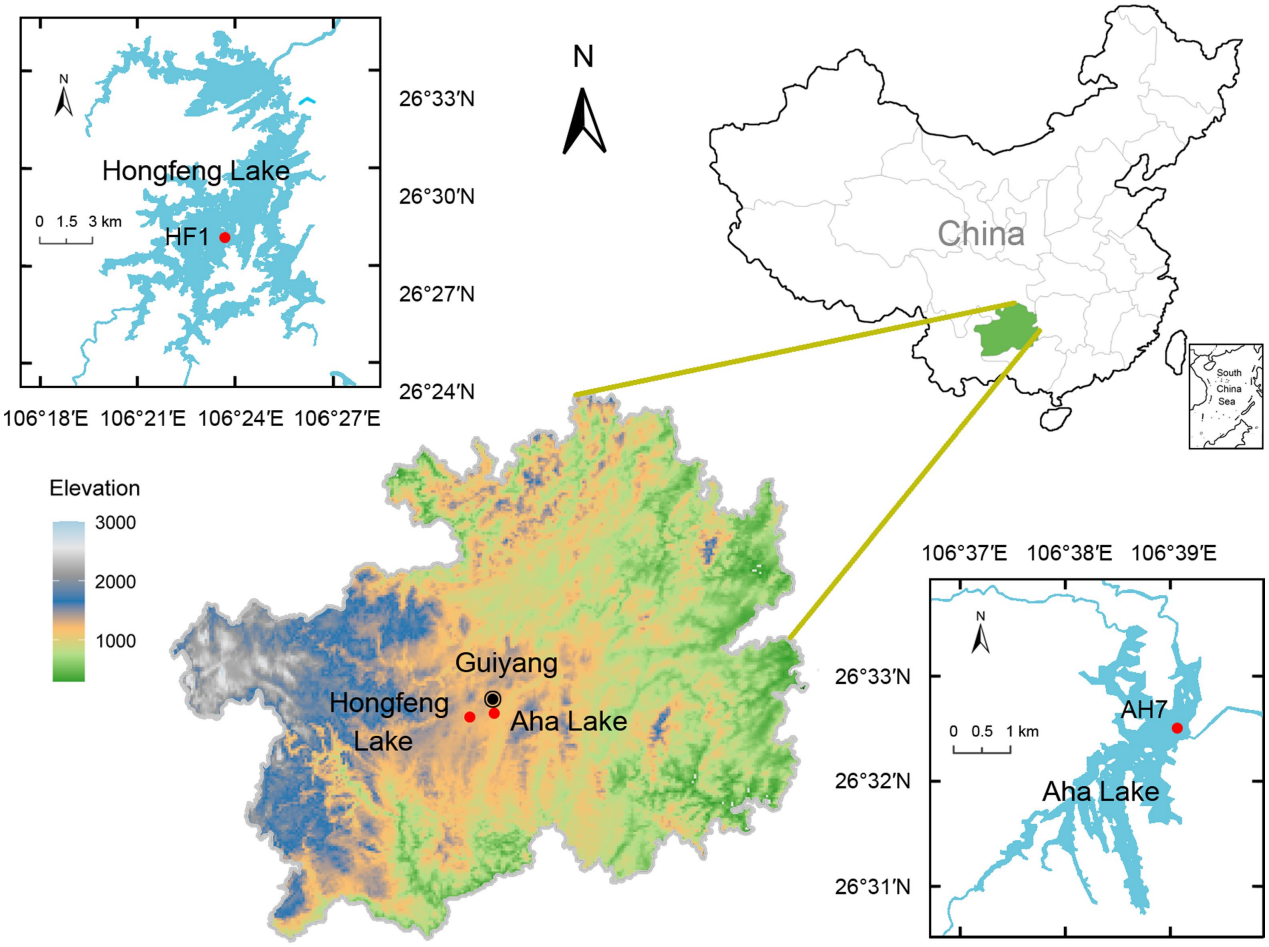
Study area and sampling location.

As artificial karst lakes, both Hongfeng and Aha Lakes have frequently experienced severe eutrophication, thereby providing an ideal setting to study P fractions in sediments. Based on the profile change of sediment P, sediment core can effectively reflect the historical activities and human disturbance of a lake. Given that suspended particles form sediment profiles through sedimentation, large amounts of water P are buried in the sediments. Meanwhile, microbes ingest the nutrients such as phosphorus from the sediment to sustain growth. Using a gravitational sampler and a polyethylene tube, we obtained two sediment cores (i.e., HF1 and AH7, Figure 1) of 62 and 60 cm lengths from Hongfeng and Aha Lakes, respectively. With water depths of 15 and 22 m, HF1 and AH7 were collected in April and June 2022, respectively. The two cores were divided into 2 cm-long sediment samples *in situ*. Each sample was thoroughly stirred and homogenized, and then placed in two 20 mL sterile bottles. To avoid cross-contamination, one sterilized spoon is used for each sample when stirring. Notably, such samples must be transferred to laboratory at −20°C within an hour. For the 61 obtained samples, one bottle was stored at −80°C for biological analysis, and the other was freeze-dried and then stored at −20°C for physicochemical analysis.

### Phosphorus fractions

The P fractions were measured by Psenner’s sequential extraction scheme as modified by Hupfer et al. (1995). This scheme has been widely used in the field of lakes to study the P fractions in sediments and provides a theoretical basis for evaluating the endogenous release of P cycling. It generally divides sediment P into five fractions, that is, loosely adsorbed P (NH_4_Cl-P), reductant-soluble P (BD-P), metal oxide-bound P (NaOH-P), calcium-bound P (HCl-P) and residual P (Res-P). BD-P refers to iron-bound P and NaOH-P indicates aluminum-bound P. It should be noted that the whole procedure was conducted with 0.20 g freeze-dried sediment for each sample. Briefly, such sediment was sequentially extracted with 1 M NH_4_Cl for 0.5 h, 0.11 M NaHCO_3_/Na_2_S_2_O_4_ for 1 h, 1 M NaOH for 16 h and 0.5 M HCl for 16 h on a thermostatic shaker (220 r min^−1^). After which, all the extracts were centrifuged at 4000 r min^−1^ for 15 min. Notably, these extracts must be filtered through a 0.45 μm polyethersulfone (PES) membrane (Jinteng, China) and then transferred to a 25 mL colorimetric tube. More importantly, when such extract with NaOH or HCl was diluted to 22 mL, its pH must be adjusted to neutral before determining the content of P fraction. Furthermore, these final residues were collected, burned at 500°C for 2 h and then shaken with 1 M HCl for 16 h to obtain the supernatant after centrifugation and filtration. Subsequently, the amount of PO_4_^3−^ was quantified with the molybdate blue method (Murphy and Riley, 1962).

Such P fractions contain three forms: organic (Po), inorganic (Pi) and total P (TP). Based on the extraction procedure above, we directly determined the loosely adsorbed Pi (NH_4_Cl-Pi), reductant-soluble Pi (BD-Pi), metal oxide-bound Pi (NaOH-Pi), calcium-bound Pi (HCl-Pi) and residual TP (Res-TP). Meanwhile, each extract was digested with 50 g L^−1^ K_2_S_2_O_8_ at 120°C for 0.5 h to obtain their corresponding TP, that is, NH_4_Cl-TP, BD-TP, NaOH-TP and HCl-TP. Then, organic P fractions NH_4_Cl-Po, BD-Po, NaOH-Po and HCl-Po were calculated according to the difference between TP and Pi. Similarly, we obtained sediment total phosphorus (STP) based on the sum of the above total P fractions.

### DNA sequencing and community analyses

Total DNA from 0.30 g sediment sample was extracted in triplicate with the MagaBio Soil/Feces Genomic DNA Purification Kit (Bioer, Hangzhou, China) based on the manufacturer’s protocol. The DNA purity or concentrations were determined by spectrophotometry (NanoDrop One, United States). Then, the three DNA samples are thoroughly mixed before the polymerase chain reaction (PCR). To amplify the V4 regions of the bacterial and archaeal *16S rRNA* gene, we selected the universal primer pairs 515F (5′-GTGYCAGCMGCCGCGGTAA-3′) and 806R (5′-GGACTACNVGGGTWTCTAAT-3′) to perform the PCR (Apprill et al., 2015; Parada et al., 2016). The reaction conditions were as follows: 94°C for 5 min, followed by 30 cycles of 94°C for 30 s, 52°C for 30 s and 72°C for 30 s, all followed by a final elongation at 72°C for 10 min and termination of the reaction at 4°C. These PCR products were mixed at an equal density ratio and then purified with E.Z.N.A. Gel Extraction Kit (Omega, United States). Sequencing was conducted on the Illumina Nova6000 platform (Illumina Inc., CA, United States) to generate 250 bp paired-end reads.

For the raw data, the barcode sequence was separated and then removed from both ends of the sequence through the R-script written by Liu et al. (2021). According to the primer sequence, the reverse sequence was transposed to the forward direction. Such sequences were then processed with a pipeline combining USEARCH 11.0 and QIIME2. The high-quality reads were screened with the default values in USEARCH, and then binned into amplicon sequence variants (ASVs) after denoising by unoise3 (Edgar and Flyvbjerg, 2015). The taxonomic identification of ASVs for the sequences was assigned using the Ribosomal Database Project (RDP; http://rdp.cme.msu.edu/) classifier algorithm with a confidence threshold of 97%, and the feature table was generated for community analysis. The above analyses were generally assisted by Liu’s script (Liu et al., 2021). Based on the frequency of the minimum reads, bacterial and archaeal communities were rarefied at 49,000 and 100 sequences for Hongfeng Lake and 100,000 and 400 sequences for Aha Lake, respectively. These sequencing data have been deposited in the NCBI Sequence Read Achieve (SRA) under accession number PRJNA885467.

### Statistical analyses

We first calculated the Sørensen dissimilarity index to indicate total beta diversity and decomposed it into species turnover and nestedness using package “betapart” in R (Baselga and Orme, 2012). According to Baselga’s framework, species turnover and nestedness were employed with Simpson and nestedness coefficients, respectively. The sediment depth distances between the samples were then computed using the Euclidean distance. Subsequently, we examined the sediment-depth patterns for bacterial and archaeal beta diversity in both lakes. Such relationships were modeled with linear or quadratic models, and the significance was estimated by Mantel test (9,999 permutations). Moreover, for these P fractions, we also investigated the variation of organic, inorganic and total P fractions along sediment depth in both lakes.

Second, to investigate the influence of sediment P forms on microbial community compositions, we performed Spearman correlation analysis for the relative abundance of the top 30 bacterial genera or top 7 archaeal genera and P fractions. Such relationships were then clustered using an unweighted pair group method with arithmetic mean (UPGMA) on Euclidean distances. Furthermore, the Mantel test (Mantel, 1967) was conducted for bacteria and archaea to reveal the relationships between beta diversity and each P fraction. In addition, we also applied the Mantel test to examine such associations among bacteria and archaea in terms of beta diversity components, and conducted linear regressions to visualize their trends.

Third, to exclude the strong collinearity between all P fractions, we applied the varclus procedure of the Hmisc R package to detect the redundancy of the variables (Wang X.-B. et al., 2017). Notably, only one variable was preserved when a high correlation (Spearman *ρ*^2^ > 0.8) was observed between these P fractions. Hence, as shown in Supplementary Figure S5, we removed NH_4_Cl-Po, HCl-TP, NaOH-TP and BD-Pi from the datasets of Hongfeng and Aha Lakes. And then, using the MRM() function of the ecodist R package (Goslee and Urban, 2007), we performed multiple regression on distance matrices (MRM) (Lichstein, 2007) to quantify such association among beta diversity components and all P fractions. For such P fractions, the influence of multicollinearity must be excluded before applying MRM.

Finally, we conducted variation partitioning analysis (VPA) (Anderson and Cribble, 1998) to identify the relative explanatory power of organic, inorganic and total P fractions and their interaction on variation in total beta diversity and its components. For the two microbial taxonomic groups, forward selection (9,999 permutations) against such biological characteristic data was performed to select the significant variables before running VPA. All of the above analyses were implemented in R V4.2.1 with packages vegan V2.5-5 (Oksanen et al., 2013), betapart V1.5.1 (Baselga et al., 2018), ecodist V2.0.1 (Goslee and Urban, 2007) and Hmisc (Harrell, 2019).

## Results

### Vertical variation of bacterial or archaeal beta diversity and P fractions

**Figure 2:**
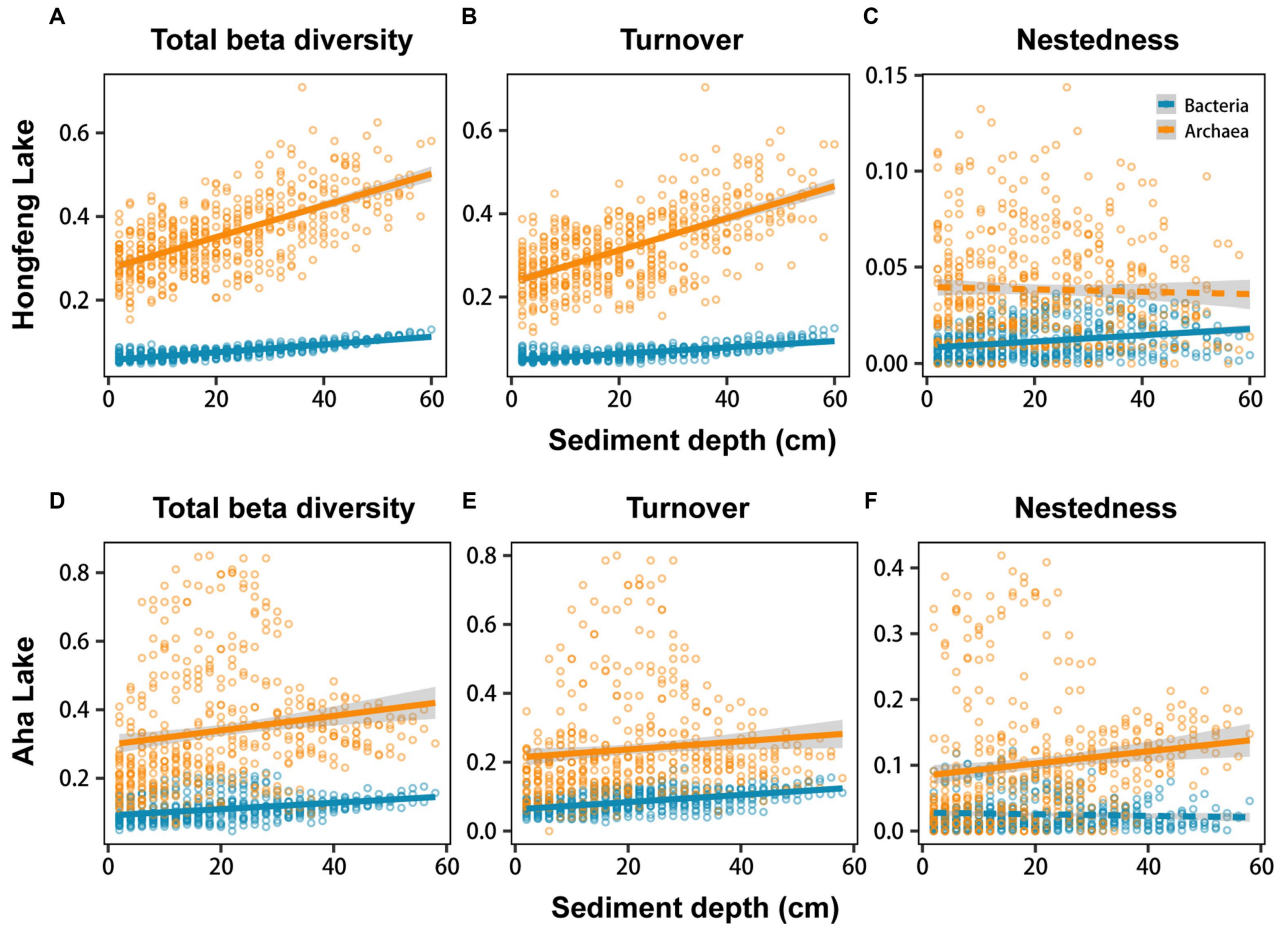
Sediment-depth patterns of bacterial and archaeal beta diversity in Hongfeng Lake **(A–C)** and Aha Lake **(D–F)**. The solid line indicates a significant relationship. More details for these models can be observed in Supplementary Table S1.

**Figure 3:**
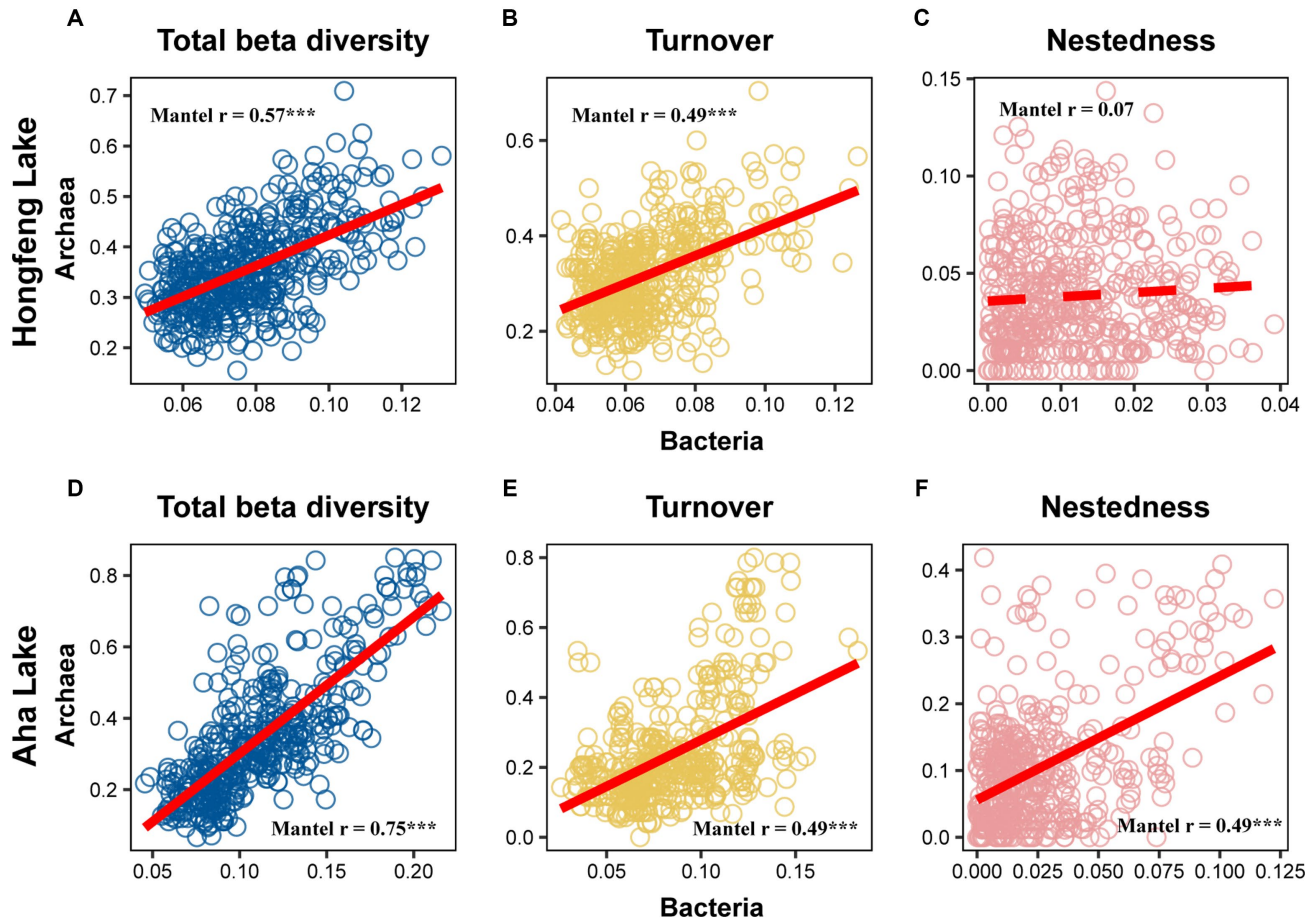
Correlation between bacterial and archaeal beta diversity in Hongfeng Lake **(A–C)** and Aha Lake **(D–F)**. The solid line indicates significant relationship. ^*^p ≤ 0.05; ^**^p < 0.01; ^***^p < 0.001.

Relationship among sediment depth and beta diversity was generally significant for bacteria or archaea in both lakes, as determined by *F*-test (Figure 2). For total beta diversity, bacteria or archaea had a significant upward trend toward deeper sediment layers. Compared to bacteria (HF: slope = 0.0009, AH: slope = 0.0009), archaea showed faster variation for total beta diversity in Hongfeng and Aha Lakes, with slopes of 0.0038 and 0.0021, respectively (Figures 2A,D and Supplementary Table S1). Similarly, archaea changed faster for species turnover than bacteria in Hongfeng Lake, with slopes of 0.0039 and 0.0008, respectively (Figure 2B and Supplementary Table S1). Significant sediment-depth pattern of species turnover was also observed in Aha Lake, but the rates of bacterial and archaeal changes were similar, with slopes of 0.0011 and 0.0012, respectively (Figure 2E and Supplementary Table S1). For species nestedness (differences in species richness), bacteria showed a significant positive relationship with sediment depth in Hongfeng Lake (Figure 2C), while archaea had an increasing (*p* < 0.05) depth-related pattern in Aha Lake (Figure 2F). Additionally, for alpha diversity, bacteria and archaea generally showed significant (*p* < 0.05) U-shaped or hump-shaped patterns along sediment-depth in both lakes (Supplementary Figure S6). In Hongfeng Lake, bacterial richness increased first and then decreased with sediment depth, while archaeal richness increased exponentially (Supplementary Figures S6A,D). For richness, evenness and Simpson diversity, whilst bacteria had significant (*p* < 0.05) depth-related patterns, there was little variation in Hongfeng Lake (Supplementary Figures S6A–C). Moreover, for evenness and Simpson diversity, bacteria decreased rapidly along sediment depth, while archaea increased slowly (Supplementary Figures S6B,C,E,F). Positive correlations among bacteria and archaea in terms of total beta diversity and its components were significantly assessed using Mantel test. For total beta diversity, bacteria and archaea showed the strongest cross-taxon congruence (Hongfeng: Mantel *r* = 0.57, *p* < 0.001; Aha: Mantel *r* = 0.75, *p* < 0.001, Figures 3A,D). Additionally, for species turnover, bacteria were significantly positively correlated with archaea (Hongfeng: Mantel *r* = 0.49, *p* < 0.001; Aha: Mantel *r* = 0.49, *p* < 0.001, Figures 3B,E). Notably, for species nestedness, bacteria had a high significant positive correlation (Mantel *r* = 0.49, *p* < 0.001) with archaea in Aha Lake, while weak association (Mantel *r* = 0.07, *p* > 0.05) in Hongfeng Lake (Figures 3C,F).

Generally, the content profiles of P fractions indicated that each P form had a decreasing trend toward deep sediments (Supplementary Figure S1). For total P fractions, BD-TP was relatively stable and declined from 264.79 μg g^−1^ at 0–2 cm to 110.19 μg g^−1^ at 60–62 cm depth in Hongfeng Lake, while it decreased sharply with sediment depth in 24–26 cm layers in Aha Lake (Supplementary Figures S1B,F). In addition, such decreasing trend was observed for NH_4_Cl-TP in Hongfeng and Aha Lakes, with ranges of 3.44–27.53 and 1.59–35.14 μg g^−1^, respectively (Supplementary Figures S1A,E). For Pi fractions, NaOH-Pi first increased greatly with depth in the 0–26 cm layers and then declined rapidly at depths greater than 46 cm in Hongfeng Lake, whereas it was relatively low (ranged at 138.15–838.55 μg g^−1^) in the 12–32 cm layers and had a general increasing trend in Aha Lake (Supplementary Figures S1C,G). Conversely, HCl-Pi declined rapidly with increasing sediment in Hongfeng Lake, and first declined greatly and then increased slightly in Aha Lake (Supplementary Figures S1D,H). For Po fractions, BD-Po showed a decreasing trend and declined from 124.35 μg g^−1^ at 0–2 cm to 8.37 μg g^−1^ at a 52–54 cm depth in Hongfeng Lake, while it changed slightly below 10 cm depth and then decreased sharply at depths greater than 12 cm in Aha Lake (Supplementary Figures S1B,F). Additionally, for NH_4_Cl-P, BD-P, NaOH-P and HCl-P, the difference in organic (i.e., NH_4_Cl-Po, BD-Po, NaOH-Po and HCl-Po), inorganic (NH_4_Cl-Pi, BD-Pi, NaOH-Pi and HCl-Pi) and total P (NH_4_Cl-TP, BD-TP, NaOH-TP and HCl-TP) forms was significantly (*p* < 0.05) observed between the two lakes (Supplementary Figure S2).

### Linkages of microbial compositions and beta diversity with P fractions

**Figure 4:**
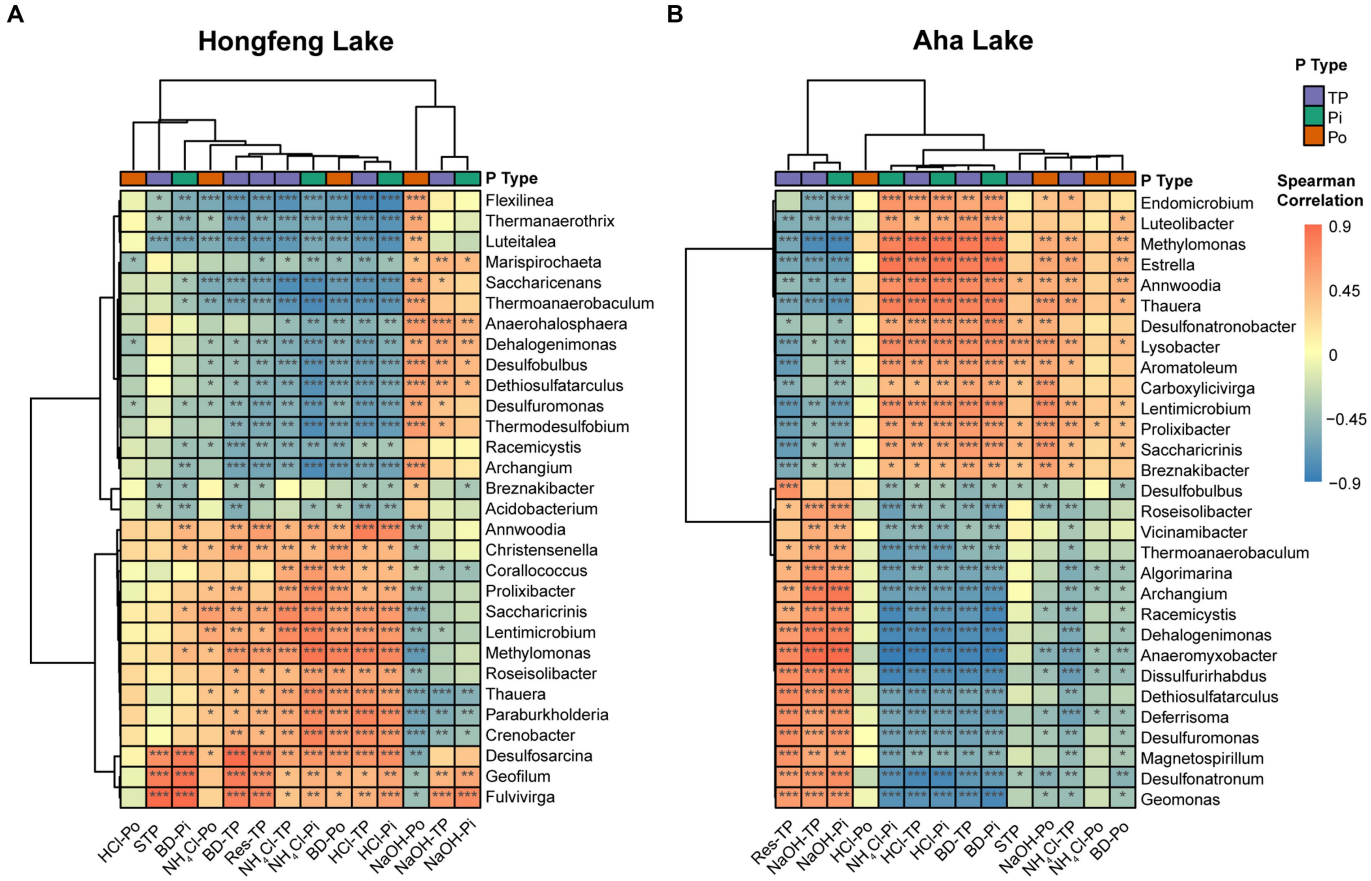
Cluster analysis of bacterial communities and P fractions at the genus level in Hongfeng Lake **(A)** and Aha Lake **(B)**. The heatmaps show the Spearman correlationship between the relative abundance of the top 30 genera and P fractions. Red denotes a positive relationship and blue indicates a negative correlation. ^***^p < 0.001, ^**^p < 0.01, ^*^p < 0.05.

Based on the Spearman correlation, strong association between bacterial or archaeal composition and P fractions was observed in both lakes (Figure 4; Supplementary Figure S3). For bacterial compositions, the total or inorganic P fractions such as NH_4_Cl-TP, NH_4_Cl-Pi, HCl-TP, and HCl-Pi had strong (*p* < 0.05) positive correlations with the dominant genera such as *Lentimicrobium* and *Saccharicrinis*, and significant (*p* < 0.05) negative correlations with other dominant genera like *Dethiosulfatarculus* and *Archangium* in both lakes (Figure 4). Additionally, organic P fractions such as BD-Po, NaOH-Po and NH_4_Cl-Po were strongly correlated with such dominant genera while HCl-Po showed weak correlations in Hongfeng Lake (Figure 4A). Similarly, there was a weak and nonsignificant relationship between HCl-Po and bacterial genera in Aha Lake (Figure 4B). Generally, compared to organic P fractions, the total or inorganic P fractions had stronger correlations with bacterial compositions in Aha Lake (Figure 4B). For archaeal compositions, *Methanothermobacter* and *Methanosalsum* are the dominant genera governing the variation of communities along a sediment depth gradient. As a mesophilic Fe(III)-reducing microorganisms, *Methanothermobacter* can couple oxidation of organic matters (e.g., Po) with the reduction of structural iron. *Methanosalsum* commonly inhabit the hypersaline alkaline lakes, and are the obligately anaerobic high salt-tolerant and alkaliphilic euryarchaea. *Methanosalsum* can regulate or provide an alkaline environment to promote the combination of Fe, Al, and Ca ions with phosphate to form insoluble P fractions. In Hongfeng Lake, the total or inorganic P fractions such as BD-TP, BD-Pi, NH_4_Cl-TP, NH_4_Cl-Pi, HCl-TP and HCl-Pi were positively correlated with *Methanothermobacter* and *Methanosalsum*, while negatively correlated with *Thermofilum* and *Thermogladius* (Supplementary Figure S3A). Moreover, BD-Po and NaOH-Po had strong correlations with the top 7 genera in Hongfeng Lake, whereas HCl-Po and NH_4_Cl-Po showed weak correlations (Supplementary Figure S3A). In Aha Lake, *Methanothermobacter* was positively correlated with total or inorganic P fractions, including HCl-TP, BD-TP, NH_4_Cl-TP, HCl-Pi, BD-Pi and NH_4_Cl-Pi, and negatively correlated with NaOH-TP and NaOH-Pi (Supplementary Figure S3B). Conversely, *Methanosalsum* showed negative correlations with HCl-TP, BD-TP, NH_4_Cl-TP, HCl-Pi, BD-Pi, and NH_4_Cl-Pi, whereas it was positively correlated with NaOH-TP and NaOH-Pi (Supplementary Figure S3B).

Furthermore, for both microbial taxa, Mantel test indicated that total beta diversity and its components also had strong correlations with P fractions (Supplementary Figure S4). For bacteria, total beta diversity or turnover were positively correlated with NH_4_Cl-Pi (Mantel *r* = 0.43 and 0.32) and BD-Po (Mantel *r* = 0.42 and 0.35) in Hongfeng Lake, and with NH_4_Cl-Pi (Mantel *r* = 0.46 and 0.50), BD-TP (Mantel *r* = 0.42 and 0.55) and HCl-Pi (Mantel *r* = 0.51 and 0.46) in Aha Lake (Supplementary Figure S4). Note that the nestedness component had strong correlations with BD-TP (Mantel *r* = 0.47) and BD-Pi (Mantel *r* = 0.52) in Hongfeng Lake (Supplementary Figure S4). For archaea, the total beta diversity or turnover showed significant positive correlations with NH_4_Cl-Pi (Mantel *r* = 0.44 and 0.38), BD-TP (Mantel *r* = 0.38 and 0.40), HCl-Pi (Mantel *r* = 0.36 and 0.40) and Res-TP (Mantel *r* = 0.40 and 0.44) in Hongfeng Lake, and with BD-TP (Mantel *r* = 0.44 and 0.53), NaOH-Pi (Mantel *r* = 0.47 and 0.46) and HCl-Pi (Mantel *r* = 0.49 and 0.48) in Aha Lake (Supplementary Figure S4). Collectively, for bacteria and archaea, both total beta diversity and species turnover are strongly correlated with P fractions.

### Effects of P fractions on bacterial and archaeal beta diversity

**Figure 5:**
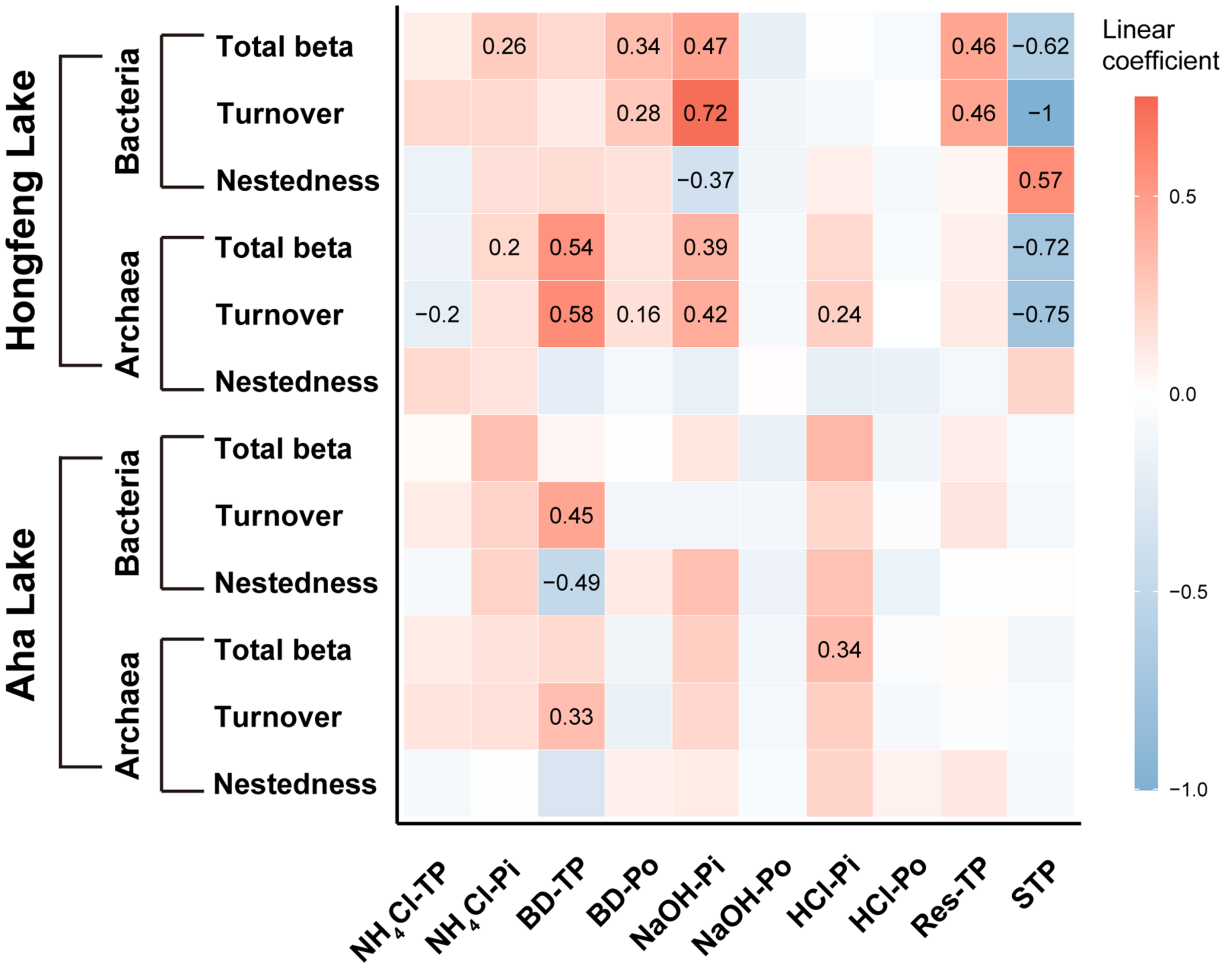
Effects of phosphorus fractions on bacterial and archaeal beta diversity. The P fractions include organic, inorganic and total P fractions. Significant relationships are visualized with squares showing numerical value. Color and value indicate the linear coefficient.

Based on the MRM (multiple regression on distance matrices) analyses, the importance of each P fraction to beta diversity varied across microbial taxa (Figure 5). For bacteria, compared to Aha Lake, NaOH-Pi and sediment total P contributed the relatively larger linear coefficient (i.e., effect size) to explain total beta diversity (linear coefficient = 0.47 and − 0.62), species turnover (linear coefficient = 0.72 and − 1.00) and nestedness (linear coefficient = −0.37 and 0.57, Figure 5) in Hongfeng Lake. Conversely, there was nonsignificant independent influence among total beta diversity and such P fractions in Aha Lake. Moreover, BD-TP was the most important driver of species turnover and nestedness in Aha Lake, with linear coefficients of 0.45 and −0.49, respectively (Figure 5). For archaea, BD-TP, NaOH-Pi and sediment total P showed high relative importance in explaining the total beta diversity (linear coefficient = 0.54, 0.39 and −0.72) and turnover component (linear coefficient = 0.58, 0.42 and −0.75) in Hongfeng Lake (Figure 5). Inconsistently, archaeal total beta diversity was primarily affected by HCl-Pi (linear coefficient = 0.34, *p* < 0.05) in Aha Lake, while only BD-TP (linear coefficient = 0.33, *p* < 0.05) constrained the turnover component. Notably, for archaea, P fractions had no independent influence on species nestedness in both lakes (*p* > 0.05, Figure 5).

**Figure 6:**
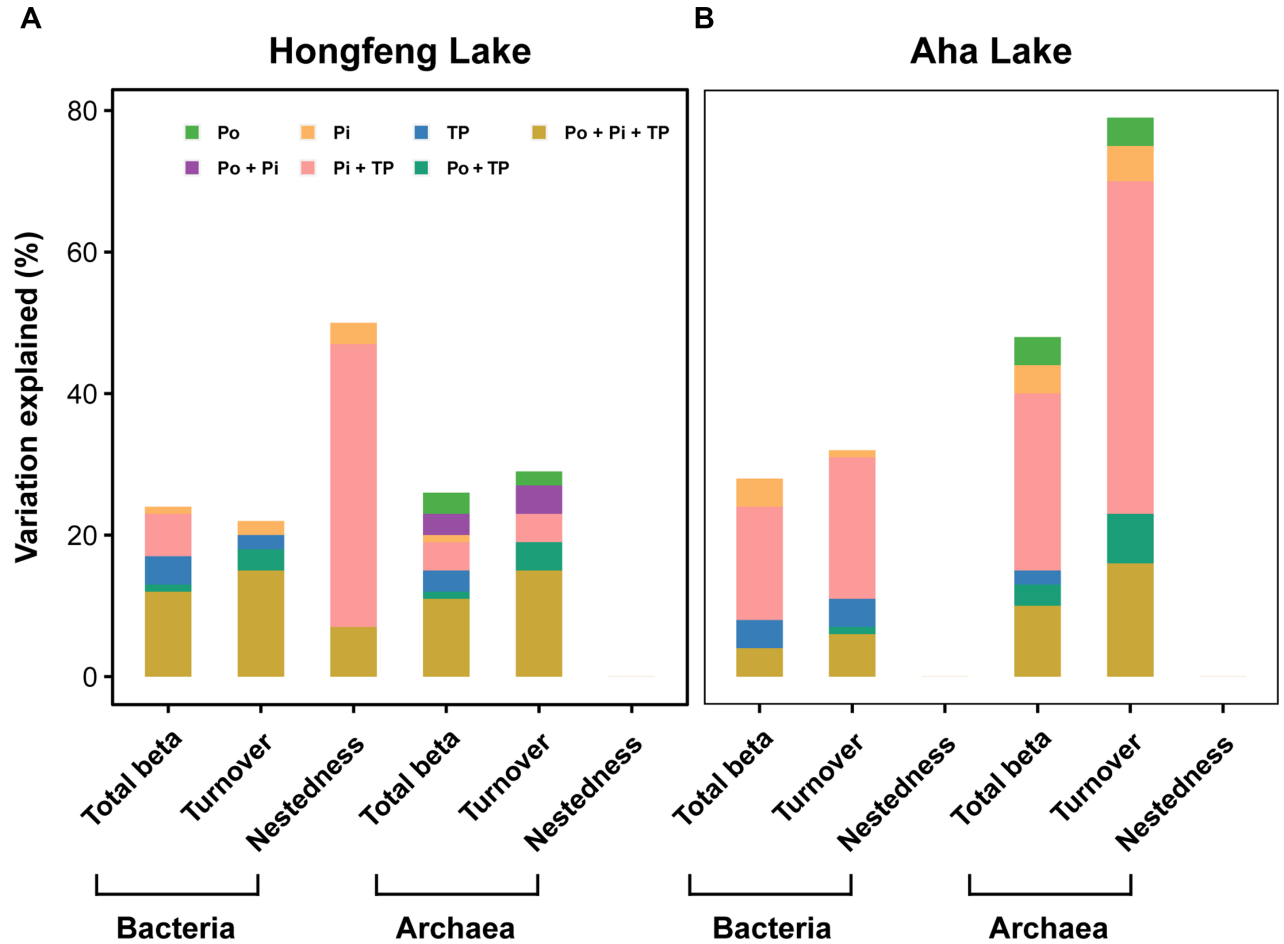
Relative importance of organic, inorganic and total P fractions in explaining variation of bacterial and archaeal beta diversity in Hongfeng Lake **(A)** and Aha Lake **(B)**. Variation partition analysis was performed to reveal the pure and joint effects of organic (Po), inorganic (Pi) and total P (TP) fractions on bacterial and archaeal beta diversity. The selected variables are shown in Supplementary Table S2. Statistical significance was evaluated using the Monte Carlo permutation test (9,999, *p* < 0.01).

In the VPAs, for bacteria or archaea, total beta diversity (*R*^2^ = 0.12 and 0.11, respectively) and turnover component (*R*^2^ = 0.15 and 0.15, respectively) were mostly explained by the joint effects of the-Po fractions, Pi fractions and TP fractions in Hongfeng Lake (Figure 6A); however, in Aha Lake, the Pi fractions and TP fractions jointly accounted for 16% and 25% of variations in the total beta diversity, while 20% and 47% of variation in the turnover component (Figure 6B). Similarly, for bacteria, the joint effects of the Pi fractions and TP fractions explained 40% of the variation in the nestedness component in Hongfeng Lake (Figure 6A). For beta diversity components, compared to Po fractions, the Pi fractions showed a greater pure effect on bacteria, while the pure effect of Po fractions on archaea was stronger than that on bacteria (Figure 6).

## Discussion

Unraveling the mechanisms underlying spatial and temporal variations in biodiversity has long been a central goal in ecology (Peters et al., 2019; Zhao et al., 2019). It has long been recognized that species turnover (i.e., species replacement) and nestedness (i.e., differences in species richness) partitioned by total beta diversity can underpin the processes shaping community structures (Zhang et al., 2020; Marta et al., 2021). For microbial succession, previous studies have mostly focused on how phosphate (i.e., PO_4_^3−^) or total P affects community variation. Much less is known, however, about the importance of P fractions to beta diversity components. To our knowledge, this study represents the first attempt to investigate how P fractions affect microbial total beta diversity and their components. We examined the variations in bacterial and archaeal beta diversity and their components along sediment depth, and further identified the relative importance of organic, inorganic and total P fractions to beta diversity. We found that (1) both bacteria and archaea had significant depth-related patterns in total beta diversity, turnover or nestedness, (2) there was taxonomic dependency among bacteria and archaea for beta diversity patterns along sediment depth, (3) the importance of each P fraction (i.e., NH_4_Cl-Pi, BD-Pi, NaOH-Pi, HCl-Pi, NH_4_Cl-Po, BD-Po, NaOH-Po, HCl-Po, NH_4_Cl-TP, BD-TP, NaOH-TP, HCl-TP and Res-TP) to total beta diversity or species turnover varied with microbial taxonomic groups, and (4) Pi fractions have higher explanation than Po fractions for bacterial beta diversity, while archaeal total beta diversity or turnover component are well explained by Po fractions.

Species turnover is the most important component contributing to total beta diversity and has been examined extensively in aquatic ecosystems (Lima et al., 2020). In this study, the total beta diversity or species turnover of bacteria and archaea increased consistently along sediment depth (Figures 2A,B,D,E), largely consolidating the predominance of species turnover. Such patterns exhibited a similar trend toward deep sediments, which aligns with the general pattern of species composition (Rooney and Azeria, 2015), where cross-taxon congruence (i.e., parallel diversity patterns among different taxa) happens if different biological groups are spatially covary in alpha or beta diversity. As expected, bacteria showed significant positive correlation (i.e., indicating parallel patterns between bacteria and archaea regarding total beta diversity and turnover) with archaea in terms of total beta diversity and turnover (Figures 3A,B,D,E), which is consistent with Yuan et al. (2022) and further underpins the taxonomical dependency between bacteria and archaea. More interestingly, such congruence indicates parallel patterns between both microbial taxa, which may be affected by similar processes like environmental selection (Shade et al., 2018). As previously reported (Specziár et al., 2018), the mechanisms responsible for species turnover are primarily triggered by environmental filtering, species competition or historical events (e.g., dispersal limitation). The intensity of selection may thus be related to environmental heterogeneity of sediment P fractions (Supplementary Figures S2, S4), which is supported by several studies addressing the importance of environmental selection on microbial communities (Yuan et al., 2021).

For bacteria or archaea, total beta diversity is generally predominated by species turnover and nestedness (Figure 2), but the species turnover explains a greater proportion of beta diversity than nestedness (Supplementary Table S1). Namely, turnover component contributes largely to total beta diversity, whereas nestedness component plays a smaller role, which is in line with previous studies on pond communities (Hill et al., 2017) and marine benthos (Bevilacqua and Terlizzi, 2020). In pond habitats, for macroinvertebrate, total beta diversity almost entirely reflects patterns of species turnover rather than nestedness. Likewise, for marine benthos, the compositional beta diversity is mainly due to species turnover, with a negligible contribution of nestedness. Species nestedness (i.e., differences in species richness caused by species gains or losses) exerts a weak influence on total beta diversity but can allow us to explicitly link species gains or losses to the mechanisms underlying compositional changes (Marta et al., 2021). For the nestedness component, our findings revealed a significant increasing depth-related pattern for bacteria in Hongfeng Lake and for archaea in Aha Lake (Figures 2C,F). The reasons for the different patterns of bacteria and archaea between the two lakes are manifold, among which is that nestedness differences stem mainly from species thinning or other ecological processes like human disturbance (Legendre, 2014). It is worth noting that species thinning may be related to intraspecific competition (i.e., a competition between individuals from the same species). Generally, competition occurs when the environmental capability supplying resources fails to meet the biological requirement, causing the organisms to interfere with each other. Intraspecific competition largely constrains the growth of organisms in populations, thereby resulting in species gain or loss (i.e., species nestedness). More specifically, intraspecific competition can lead to self-thinning, in which less-capable individuals die while more-competitive individuals survive (Begon et al., 1990). Our findings reflected that intraspecific competition affects the nestedness component through species thinning and thereby govern community composition, which is supported by strong associations between the top 30 bacterial genera or top 7 archaeal genera and P fractions (Figure 4; Supplementary Figure S3). For instance, *Geofilum* and *Fulvivirga* show significant positive correlations with BD-Pi, BD-TP, NH_4_Cl-TP, NH_4_Cl-Pi, BD-Po, HCl-TP, HCl-Pi, NaOH-TP and NaOH-Pi in Hongfeng Lake, implying that the two microbes may compete within the species for the same nutrients. Although bacteria had no significant depth-related pattern for nestedness in Aha Lake, it is not difficult to comprehend that intraspecific competition occurs within bacterial communities living in the P-limited habitat (Figure 4B).

Beta diversity is substantially governed by species pool (i.e., the set of potential species coexisting in a target community in a certain region) and could be constrained by local environmental factors. Previous studies have shown that environmental selection is the main ecological process in driving the biogeographic patterns of biodiversity (Wang J. et al., 2017). In our study, the decreasing trend of P fractions along sediment depth was generally found in NH_4_Cl-P, BD-P, NaOH-P or HCl-P (Supplementary Figure S1) and there existed significant differences between the two lakes regarding such P fractions (Supplementary Figure S2), indicating strong environmental heterogeneity between Hongfeng and Aha Lakes. Environmental heterogeneity can effectively determine the ecological processes in controlling microbial community variation in freshwater ecosystems (Huber et al., 2020). In particular, strong heterogeneous selection (i.e., selection by environmental heterogeneity), limiting the biological growth to produce community variation (i.e., beta diversity), can filter species from the species pool to increase or promote beta diversity (Zhang et al., 2020). For bacteria or archaea, we observed that total beta diversity and turnover showed strong correlations with P fractions such as NH_4_Cl-Pi and BD-Po in Hongfeng Lake, and with P fractions like BD-TP and HCl-Pi in Aha Lake (Supplementary Figure S4), largely confirming that P fractions have profound effects on biodiversity in P-limited lakes. Further, the result of the MRM analyses suggested that, environmental distance of P fractions such as NaOH-Pi, BD-TP and HCl-Pi can individually affect total beta diversity or species turnover regarding both microbial taxa (Figure 5), confirming that environmental selection has a dominant influence on the mechanisms underlying variation in bacterial or archaeal communities. As such, the depth-decay patterns of bacterial and archaeal beta diversity may be mainly attributed to the environmental selection.

Environmental factors can directly drive the variation in total beta diversity and also alter it by affecting species turnover and nestedness, thereby exerting a profound influence on the maintenance of biodiversity in aquatic or terrestrial ecosystems (Martiny et al., 2011). Thus, our findings further revealed the pure and joint effects of the organic, inorganic and total P fractions on bacterial and archaeal beta diversity. Generally, such P fractions had high explanations for bacterial or archaeal beta diversity in Hongfeng and Aha Lakes. Especially, the joint effects of inorganic and total P fractions could explain a great proportion of total beta diversity or its components (Figure 6). These results, together with those reported in previous studies (Watson et al., 2018; Pu et al., 2020; Yin et al., 2022), collectively indicate a close link between microbial community variation and P fractions, which is interesting but rarely reported in aquatic ecosystems. Moreover, for the pure effect, we found that the Pi fractions had a higher explanation than Po fractions for bacterial beta diversity (Figure 6). As an essential component of nucleic acids, lipids or storage energy molecules, Pi can substantially influence the shaping of microbial community structure and ecological patterns or processes (Zheng et al., 2021). As such, this finding largely stresses the importance of Pi in shaping bacterial community structures, which is concordant with previous findings on aquatic bacteria (Yuan et al., 2022) or phytoplankton (Guo et al., 2022). For example, in less eutrophic lowland streams, changes in soluble reactive phosphorus (i.e., Pi) between sites are the key driver homogenizing the total β-diversity of phytoplankton (Frau et al., 2023). Admittedly, Pi is an indispensable nutrient triggering the growth of aquatic microorganisms and has been widely reported in previous literature (Carvalho et al., 2011). More intriguingly, for total beta diversity or species turnover, we found that Po fractions had a higher explanation for archaea than bacteria (Figure 6), presumably owing to the anaerobic biodegradation of *Euryarchaeota*. Recent studies have suggested that sediment aeration can increase the content of reductant-soluble phosphorus fractions (Fe/Al-P) and promote the mineralization of organic phosphorus to the labile form (Dieter et al., 2015). In fact, due to the lack of oxygen content, bacterial communities are gradually dominated by anaerobic reductive bacteria toward deep sediments (Fonseca et al., 2022). In our study, *Euryarchaeota* is the dominant phylum of the archaeal community and contains large amounts of anaerobic methane oxidizing archaea such as *Methanothermobacter* and *Methanosalsum*. More intriguingly, *Methanothermobacter* was significantly (*p* < 0.05) negatively correlated with NaOH-Po in Hongfeng Lake, while *Methanosalsum* had significant (*p* < 0.05) negative correlation with NaOH-Po and BD-Po in Aha Lake (Supplementary Figure S3), indicating obvious differences in the Po mineralizing archaea between the two lakes. Such oxidizing archaea can completely mineralize organic matters like Po fractions to inorganic form under anaerobic conditions, wherein inorganic P can be well absorbed by archaea to promote their body growth (Baker et al., 2020). Furthermore, for bacteria and archaea, such effects including pure and joint effects, also indicated that P fractions could influence total beta diversity by driving its turnover component, reflecting the predominance of species turnover by environmental or spatial filters. Taken together, these findings emphasize the importance of P fractions to variations in bacterial and archaeal communities and have important implications (e.g., maintaining biodiversity by regulating nutrients like P based on the properties of P fractions) for how we protect biological diversity in aquatic ecosystems.

Nevertheless, there are two caveats to the interpretation of our studies. First, for bacteria or archaea, species nestedness may be governed by other ecological processes in Aha Lake. As previously reported (Rapacciuolo et al., 2019), strong Mantel correlation between bacteria and archaea regarding nestedness component, indicates that their parallel patterns may be driven by similar processes in Aha Lake (Figure 3F). Surprisingly, P fractions show a low explanation (i.e., P sources were abundant in sediments without limiting species gain and loss) for species nestedness in Aha Lake (Figures 5, 6), implying that their depth-related patterns may not be determined by the selection of P fractions, but may be influenced by species interaction or other environmental factors. Consistently, recent studies have suggested that species nestedness shows complex nonlinear relationships with extirpation and colonization (Lu et al., 2019), which largely determines whether beta diversity increases or decreases (Tatsumi et al., 2020). Therefore, for such unique species nestedness, extirpation and colonization considered in future theoretical works may better explain such biogeographic patterns in community variation. Note that both species turnover or nestedness and extirpation or colonization, can largely reveal the underlying mechanisms of bacterial and archaeal succession, thereby enhancing our understanding of the distribution of microbial communities and providing direct evidence for studying the function and stability of lake ecosystems.

Second, this study primarily focused on the environmental selection imposed by P fractions for microbial community variation, without considering the influence of stochastic ecological processes. It is broadly recognized that community assembly is simultaneously governed by deterministic (e.g., species-environment associations and habitat filtering) and stochastic (e.g., ecological drift and dispersal limitation) processes (Wang et al., 2013). Ecologically, deterministic selection by specific environmental variables is generally pivotal, although in some cases, stochastic or neutral processes may dominate (Stegen et al., 2012). For instance, in the Shenzhen River-Bay system, South China, environmental selection such as salinity and water temperature are pronounced for governing bacterial and archaeal community composition and assembly processes (Wang Y. et al., 2020). Likewise, such major role of deterministic processes has also been observed in soils (Moroenyane et al., 2016) and rivers (Vilmi et al., 2020). Similarly, phosphorus fractions contain large amounts of organic or inorganic P, providing essential nutrients for microbial growth (Zheng et al., 2021). Such nutrients can alter even biodiversity and its relationship with temperature in large-scale field experiments (Wang et al., 2016b). Thus, as an environmental filtering, P fractions can deterministically govern bacterial and archaeal community variation. Especially in mesotrophic lakes, controlling endogenous phosphorus release through community response of sediment microbes can effectively improve lake water quality, thereby strengthening lake conservation and management. Briefly, our study points to general rules controlling the relative contribution of species turnover and nestedness regarding bacteria and archaea, and we also encourage further works to complement such results by considering more ecological processes like deterministic and stochastic processes.

## Conclusion

In summary, our findings elucidated the underlying mechanisms of bacterial or archaeal communities along sediment depth and provided ecological insights into how eutrophication poses a threat to microbial diversity. This study for the first time revealed the importance of P fractions to the mechanism underlying microbial total beta diversity in aquatic ecosystems. For bacteria and archaea, we found significant increasing depth-related patterns in total beta diversity, species turnover or nestedness. Intriguingly, bacteria showed significant parallel patterns with archaea regarding total beta diversity and species turnover, which is largely supported by similar processes like environmental selection. For both microbial taxa, total beta diversity and species turnover were primarily constrained by NaOH-Pi and STP in Hongfeng Lake, while largely affected by BD-TP or HCl-Pi in Aha Lake. Moreover, NaOH-Pi and sediment total phosphorus can influence bacterial total beta diversity by driving species nestedness in Hongfeng Lake. The joint effects of Po fractions, Pi fractions and TP fractions indicated that the P fractions are important for bacterial and archaeal beta diversity. Compared to Po fractions, Pi fractions have greater pure effects on bacterial beta diversity. Meanwhile, Po fractions show a higher explanation for archaeal beta diversity than bacteria. To date, both partitioning beta diversity and P fractions are pervasive topics in ecology or environmental science, but they are rarely coupled to reveal the biological effects in the P cycle or the effects of the P source on biodiversity conservation. In fact, sediment microbes and P fractions determine whether the trophic state of the lake will transition from mesotrophic to eutrophication, and have crucial roles in the biogeochemical process of lake P cycling. Further studies should be encouraged to investigate the associations between P fractions and more taxonomic groups like fungi and algae in aquatic ecosystems with different trophic states (e.g., eutrophic and oligotrophic).

## Data availability statement

The datasets presented in this study can be found in online repositories. The names of the repository/repositories and accession number(s) can be found in the article/Supplementary material.

## Author contributions

HY: Conceptualization, Data curation, Formal analysis, Investigation, Methodology, Software, Validation, Visualization, Writing – original draft, Writing – review & editing. RZ: Conceptualization, Data curation, Funding acquisition, Investigation, Project administration, Resources, Supervision, Validation, Writing – review & editing. QLi: Conceptualization, Formal analysis, Investigation, Methodology, Software, Supervision, Validation, Visualization, Writing – review & editing. QH: Formal analysis, Investigation, Methodology, Project administration, Software, Writing – review & editing. QLu: Investigation, Project administration, Supervision, Validation, Writing – review & editing. JW: Data curation, Investigation, Supervision, Writing – review & editing.
