# Supplementary material for: Unveiling the ecological significance of phosphorus fractions in shaping bacterial and archaeal beta diversity in mesotrophic lakes
Source: Front Microbiol. 2023 Oct 11;14:1279751. doi: 10.3389/fmicb.2023.1279751 (PMC10598868; doi:10.3389/fmicb.2023.1279751)
Supplement: Supplementary file 1 [file Data_Sheet_1.docx]

Supplementary Material

**Table S1** **Summary of the linear or quadratic models evaluated for the depth-related patterns of total beta diversity and its components.**

| **Lake** | **Beta diversity** | **Bacteria** | | | **Archaea** | | |
| --- | --- | --- | --- | --- | --- | --- | --- |
|  |  | **R^2^** | ***P*** | **Slope** | **R^2^** | ***P*** | **Slope** |
| Hongfeng | Total beta | 0.69 | 4.6e-121 | 0.0009 | 0.40 | 7.4e-54 | 0.0038 |
|  | Turnover | 0.54 | 1.6e-80 | 0.0008 | 0.38 | 1.4e-50 | 0.0039 |
|  | Nestedness | 0.07 | 4.5e-09 | 0.0002 | 0 | 5.0e-01 | -6.2e-05 |
| Aha | Total beta | 0.14 | 3.7e-16 | 0.0009 | 0.03 | 4.0e-04 | 0.0021 |
|  | Turnover | 0.27 | 4.4e-31 | 0.0011 | 0.01 | 2.4e-02 | 0.0012 |
|  | Nestedness | 0.002 | 1.5e-01 | -0.0001 | 0.02 | 3.6e-03 | 0.0009 |

**Table S2** **P** **fractions used in variation partition analyses.** We considered three groups of explanatory variables for bacterial and archaeal community composition: organic P fractions (Po), inorganic P fractions (Pi) and total P fractions (TP). Po: NH_4_Cl-Po, BD-Po, NaOH-Po and HCl-Po; Pi: NH_4_Cl-Pi, BD-Pi, NaOH-Pi and HCl-Pi; TP: NH_4_Cl-TP, BD-TP, NaOH-TP, HCl-TP, Res-TP and STP.

| **Lake** | **Microbe** | **Response** | **Po** | **Pi** | **TP** |
| --- | --- | --- | --- | --- | --- |
| Hongfeng | Bacteria | Total beta | BD-Po^**^ | NH_4_Cl-Pi^**^ + NaOH-Pi^*^ | TP^**^ + BD-TP^**^ |
|  |  | Turnover | BD-Po^**^ + NaOH-Po | NH_4_Cl-Pi^**^ + NaOH-Pi | TP^**^ + BD-TP^**^ |
|  |  | Nestedness | NaOH-Po | HCl-Pi^**^ + NaOH-Pi^**^ | BD-TP^**^ |
|  | Archaea | Total beta | BD-Po^**^ | NH_4_Cl-Pi^**^ | Res-TP^**^ |
|  |  | Turnover | BD-Po^**^ + NaOH-Po | NH_4_Cl-Pi^**^ | Res-TP^**^ |
|  |  | Nestedness | HCl-Po | NH_4_Cl-Pi | STP |
| Aha | Bacteria | Total beta | BD-Po + NaOH-Po | HCl-Pi^*^ + NH_4_Cl-Pi | BD-TP^**^ |
|  |  | Turnover | NaOH-Po | NH_4_Cl-Pi + HCl-Pi | BD-TP^**^ |
|  |  | Nestedness | NaOH-Po | NH_4_Cl-Pi | Res-TP |
|  | Archaea | Total beta | NaOH-Po^*^ | HCl-Pi^**^ | BD-TP^**^ |
|  |  | Turnover | NaOH-Po^*^ | HCl-Pi^*^ + NH_4_Cl-Pi | BD-TP^**^ |
|  |  | Nestedness | BD-Po | NH_4_Cl-Pi | Res-TP |

^*^, *P* < 0.05; ^**^, *P* < 0.01; ^***^*P* < 0.001.


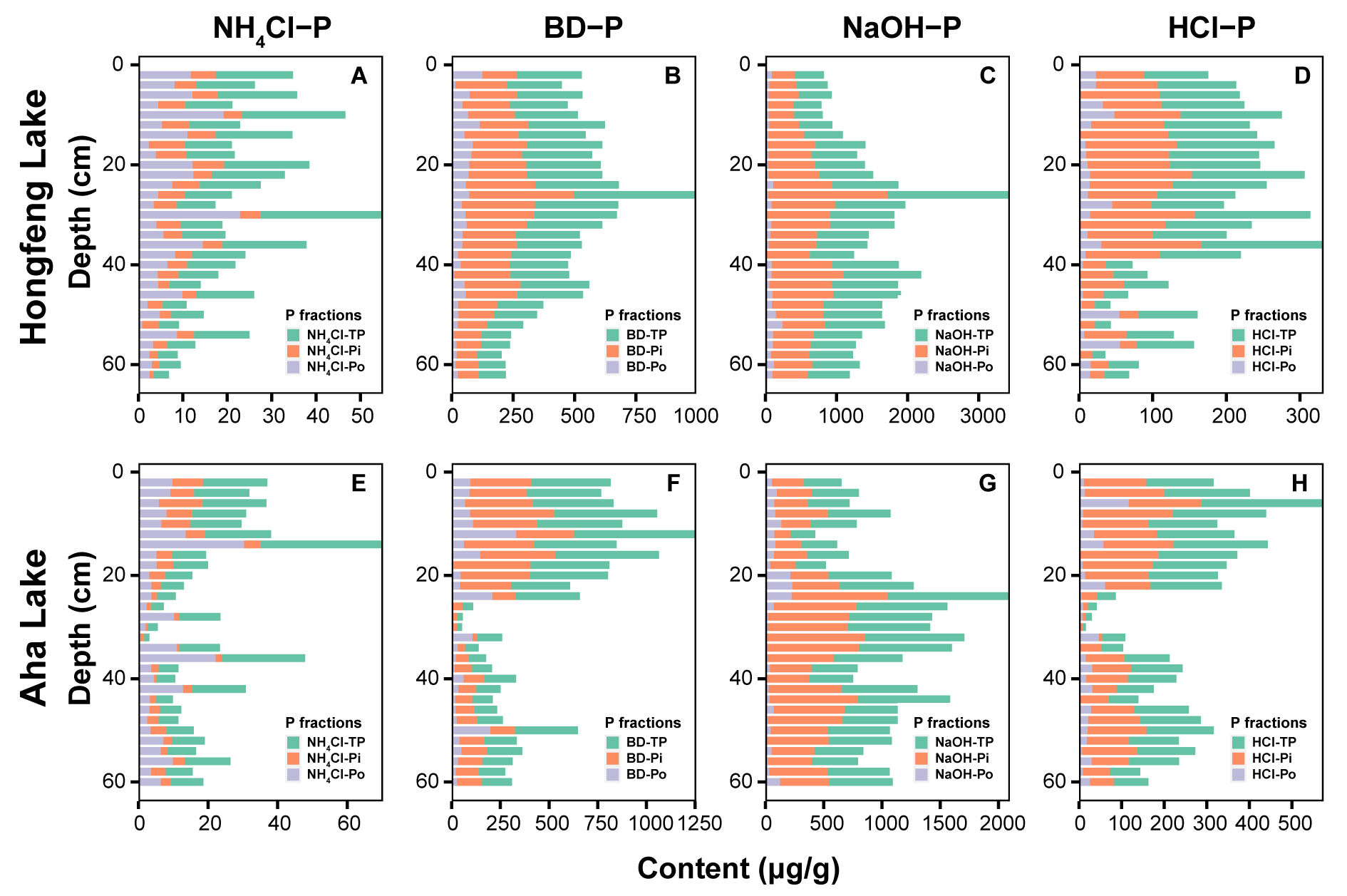


**Figure S1 Content profiles of sediment P fractions in Hongfeng (A-D) and Aha (E-H) Lakes.** The P fractions include the loosely adsorbed P (NH_4_Cl-P), reductant-soluble P (BD-P), metal oxide-bound P (NaOH-P), and calcium-bound P (HCl-P). Each P fraction is divided into organic (Po), inorganic (Pi) and total P (TP) fractions. NH_4_Cl-P: NH_4_Cl-Po, NH_4_Cl-Pi and NH_4_Cl-TP (A, E); BD-P: BD-Po, BD-Pi and BD-TP (B, F); NaOH-P: NaOH-Po, NaOH-Pi and NaOH-TP (C, G); HCl-P: HCl-Po, HCl-Pi and HCl-TP (D, H).


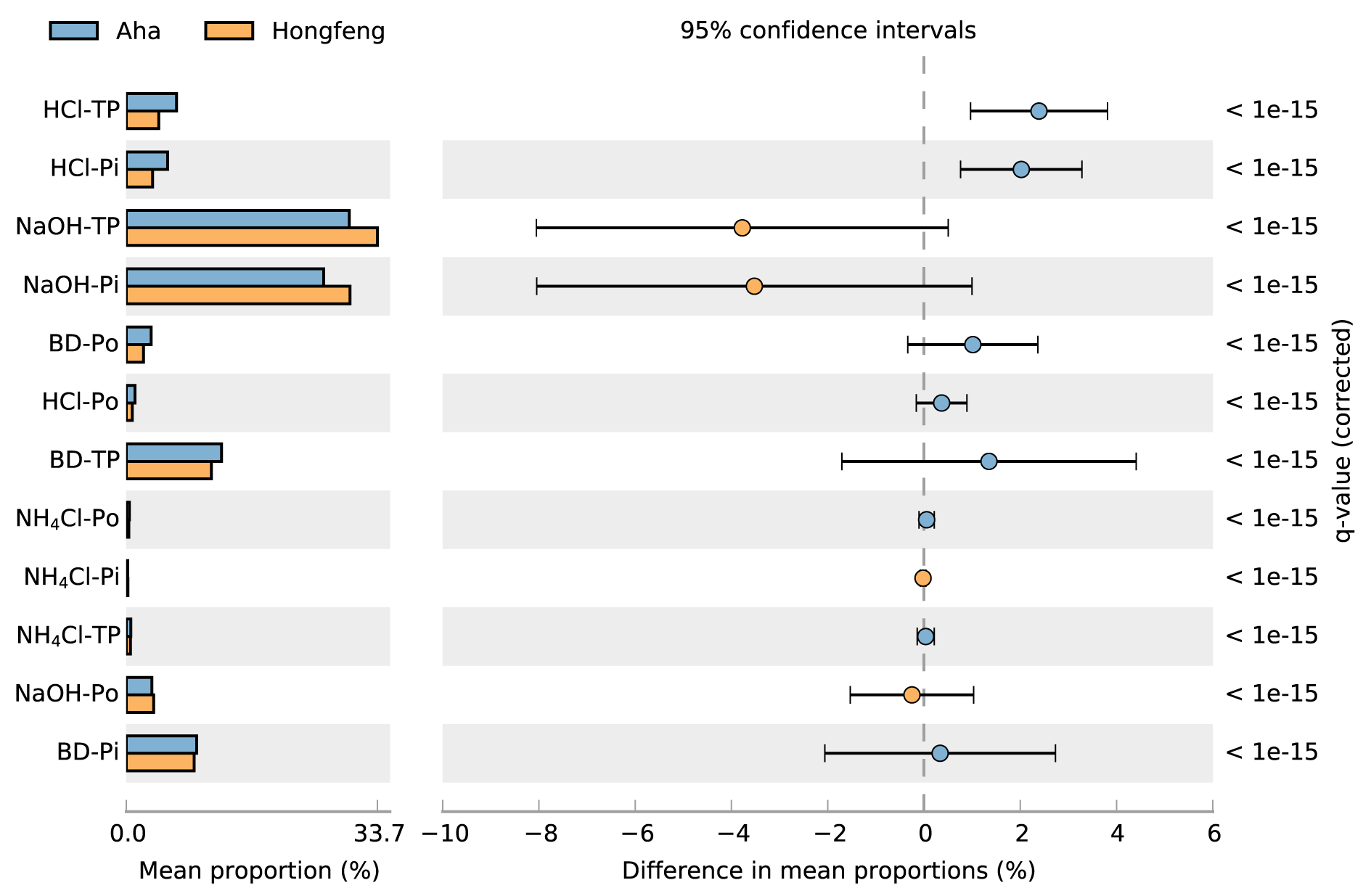


**Figure S2 Comparison of sediment P fractions between Hongfeng and Aha Lakes.**


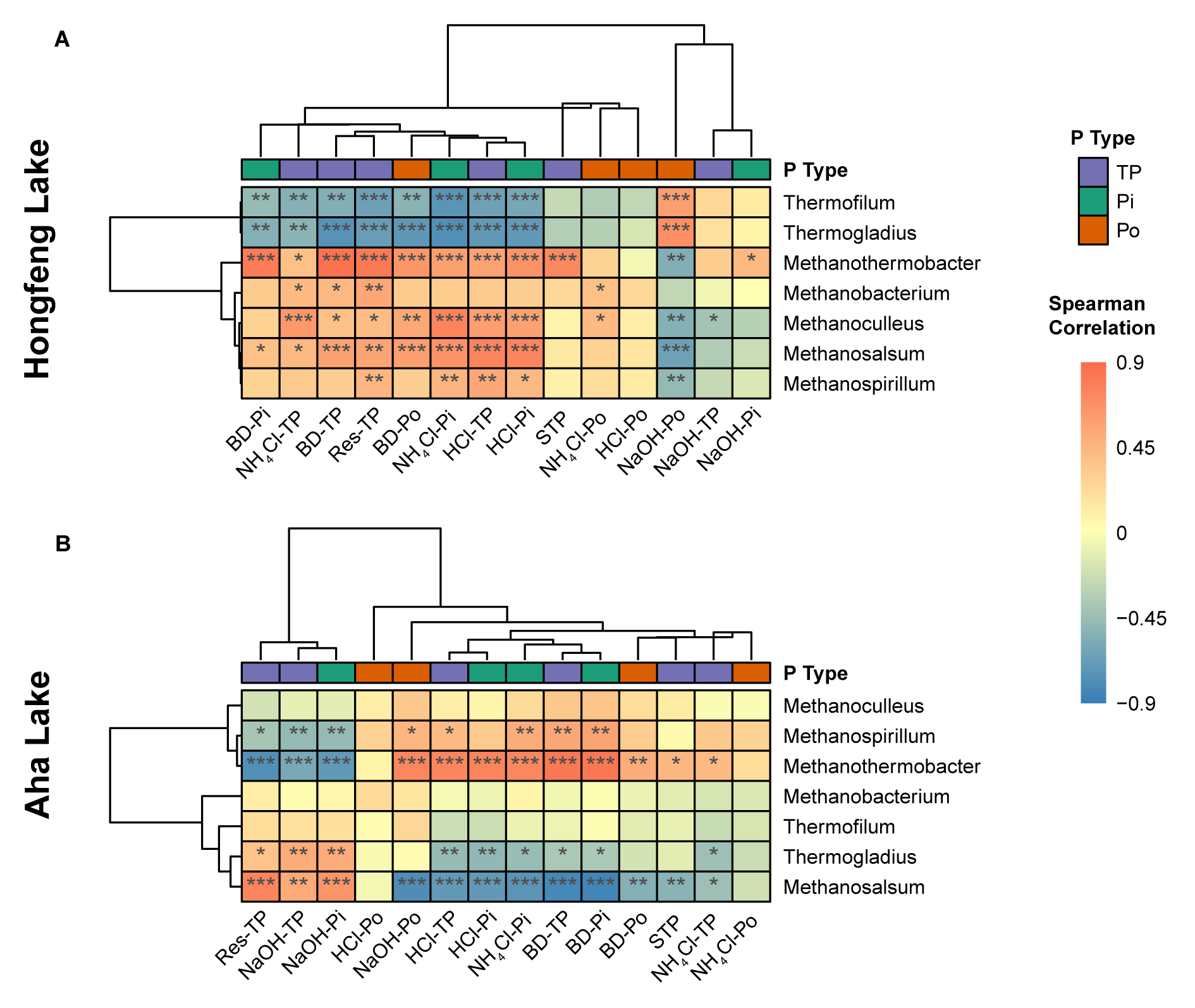


**Figure S3 Cluster analysis of archaeal communities and P fractions at the genus level.** The heatmaps show the Spearman correlationship between the relative abundance of the top 7 genera and the contents of P fractions. Red and Blue indicate positive and negative correlations, respectively. ****P* < 0.001, ***P* < 0.01, **P* < 0.05.


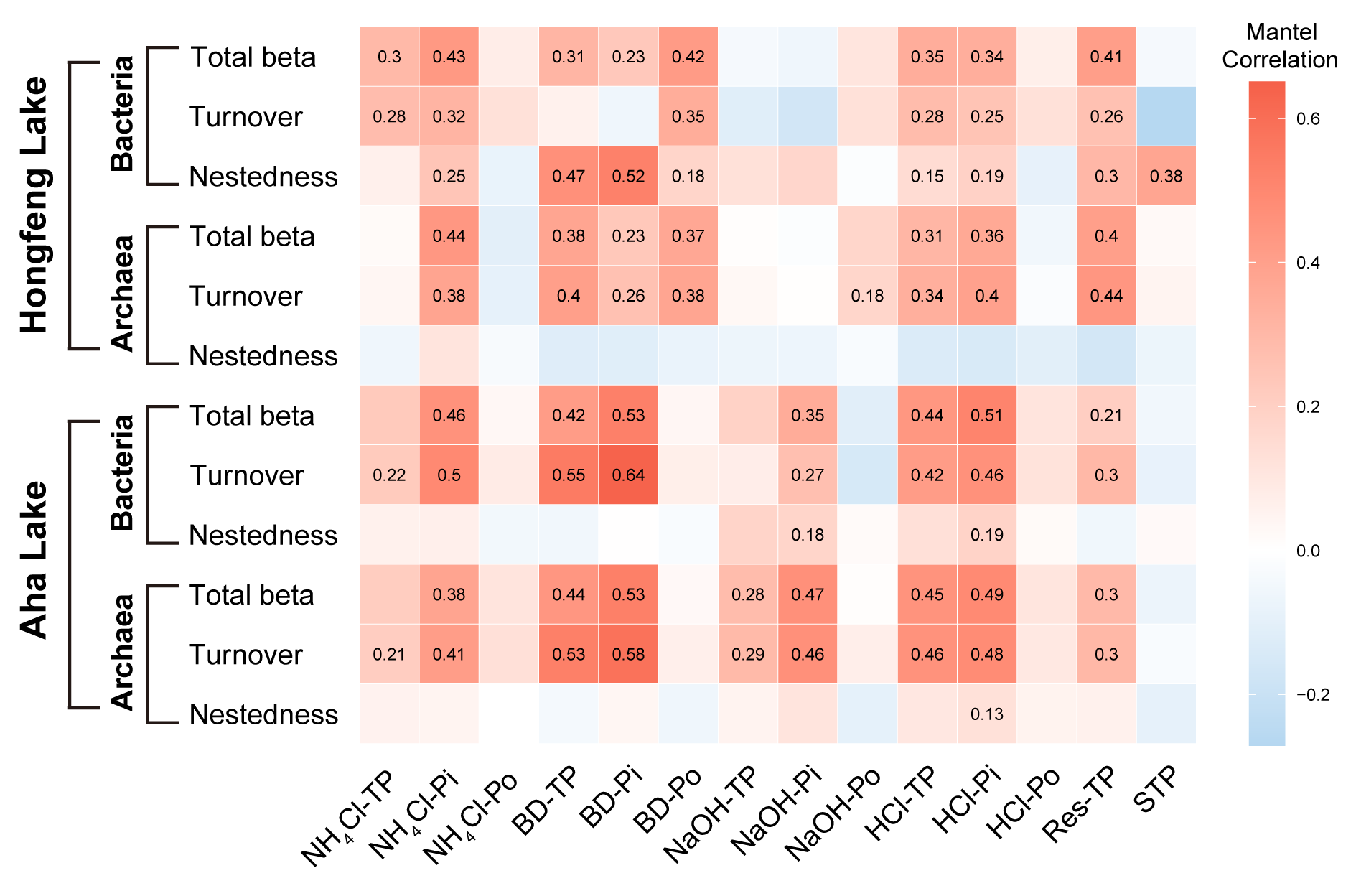


**Figure S4 Correlation between P fractions and bacterial or archaeal total beta diversity and their components.** Mantel test was performed to examine the relationships between P fractions and the total beta diversity or its two components. The value indicates the correlation. Significant (*P* < 0.05) relationships are visualized with the squares showing the numerical values.


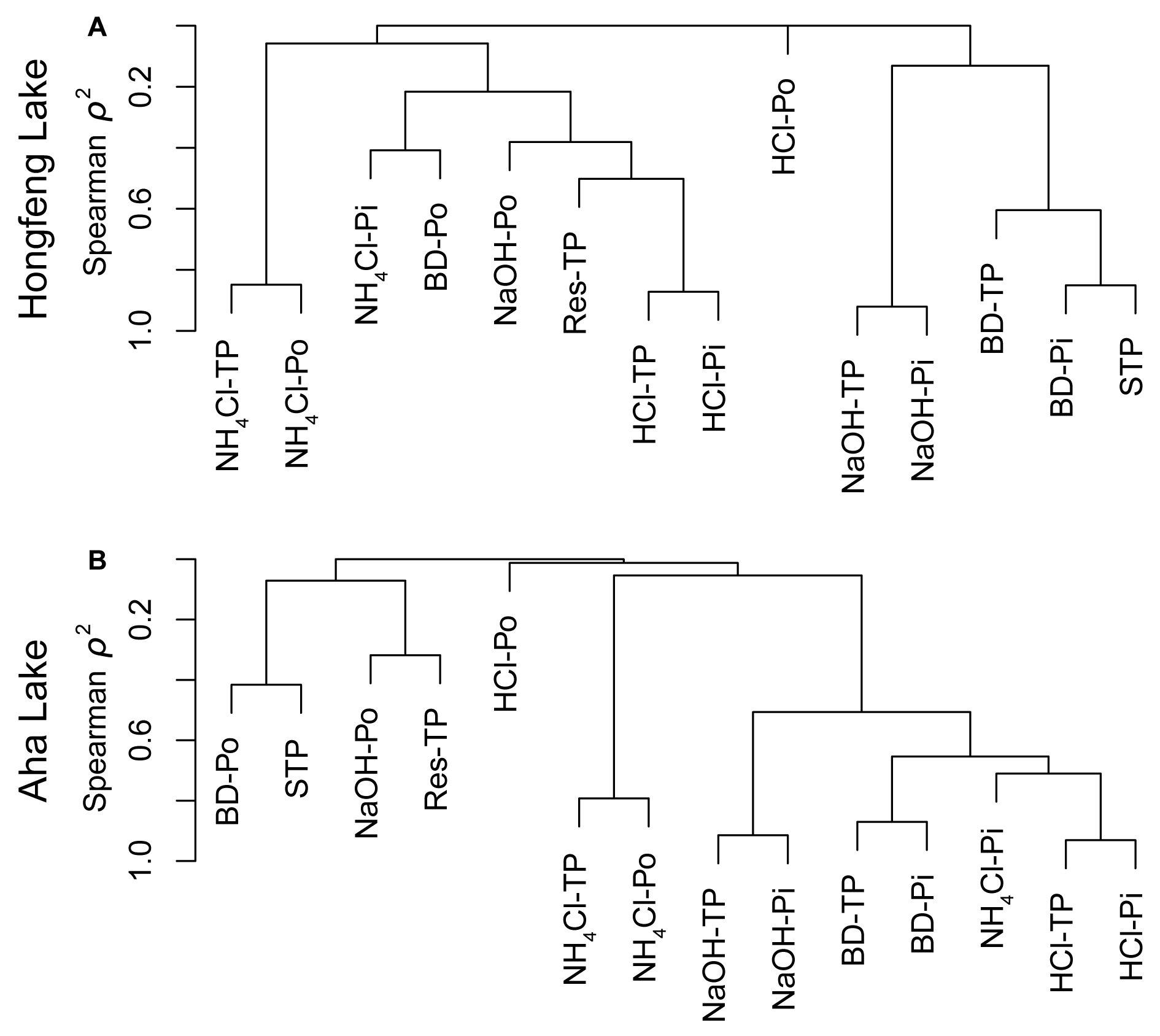


**Figure S5 Cluster analysis of the measured P fractions.** The redundancy of such P fractions is evaluated with varclus in the Hmisc R package to avoid the effects of multi-collinearity. When Spearman ρ^2^ > 0.8, only one of these variables will be retained.


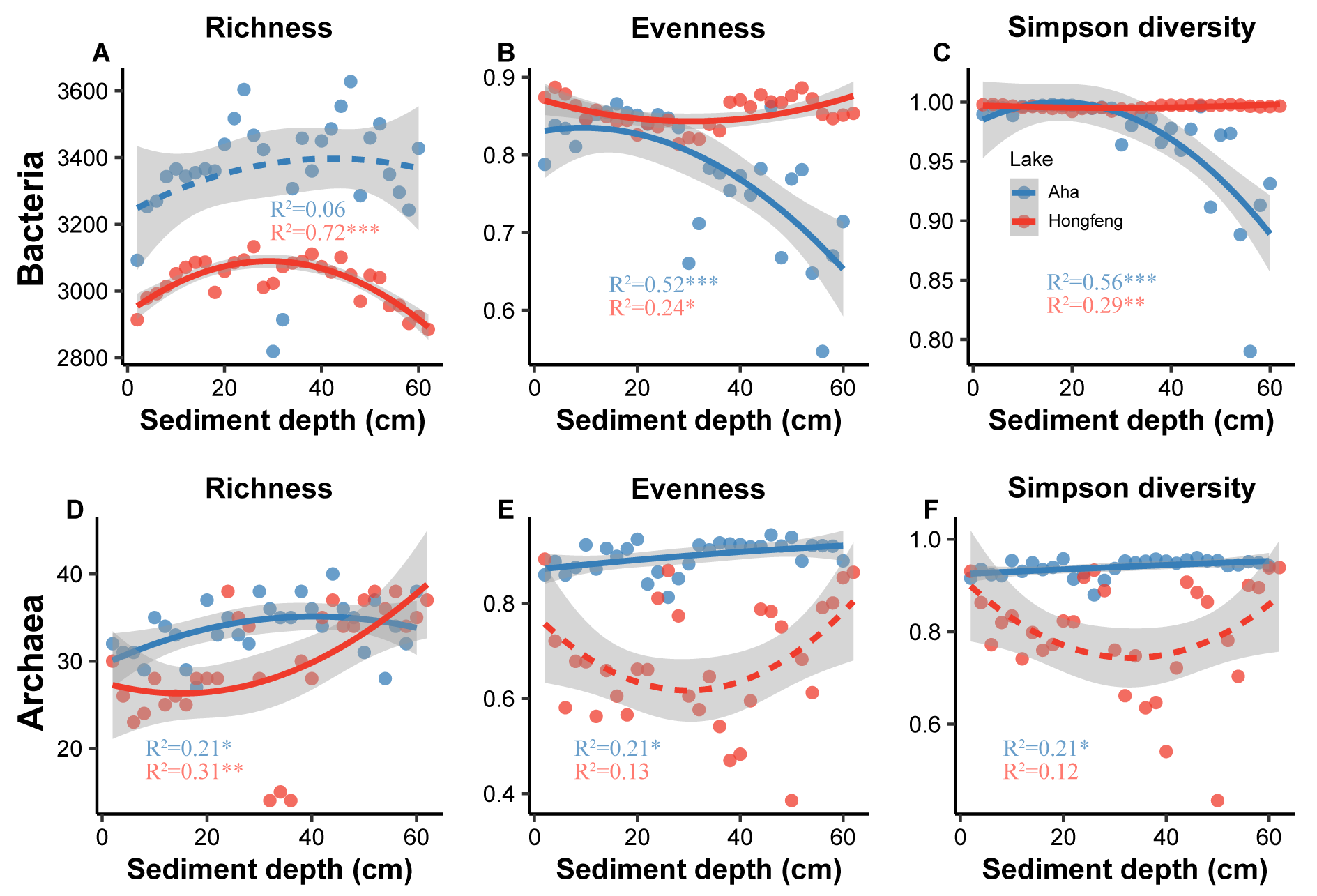


**Figure S6 Sediment-depth patterns in bacterial and archaeal alpha diversity.** The solid line indicates a significant relationship. **P* ≤ 0.05; ***P* < 0.01; ****P* < 0.001.
